# Associations Between Wearable-Specific Indicators of Physical Activity Behaviour and Insulin Sensitivity and Glycated Haemoglobin in the General Population: Results from the ORISCAV-LUX 2 Study

**DOI:** 10.1186/s40798-022-00541-9

**Published:** 2022-12-12

**Authors:** Anne Backes, Gloria A. Aguayo, Paul J. Collings, Douae El Fatouhi, Guy Fagherazzi, Laurent Malisoux, ORISCAV-LUX Study Group, Ala’a Alkerwi, Stephanie Noppe, Charles Delagardelle, Jean Beissel, Anna Chioti, Saverio Stranges, Jean-Claude Schmit, Marie-Lise Lair, Marylène D’Incau, Jessica Pastore, Gwenaëlle Le Coroller, Gloria A Aguayo, Brice Appenzeller, Sophie Couffignal, Manon Gantenbein, Yvan Devaux, Michel Vaillant, Laetitia Huiart, Dritan Bejko, Torsten Bohn, Hanen Samouda, Magali Perquin, Maria Ruiz, Isabelle Ernens

**Affiliations:** 1 Physical Activity, Sport and Health Research Group, Department of Precision Health, Luxembourg Institute of Health, 1A-B, rue Thomas Edison, L-1445 Strassen, Luxembourg; 2 Deep Digital Phenotyping Research Unit, Department of Precision Health, Luxembourg Institute of Health, Strassen, Luxembourg; 3 Exposome and Heredity Team, Center of Research in Epidemiology and Population Health, Gustave Roussy, Inserm U1018, UVSQ, Paris-Saclay University, Villejuif, France

**Keywords:** *Accelerometry*, *Wearable sensors*, *Physical activity pattern*, *Glycaemic control*

## Abstract

**Background:**

Parameters derived from an acceleration signal, such as the time accumulated in sedentary behaviour or moderate to vigorous physical activity (MVPA), may not be sufficient to describe physical activity (PA) which is a complex behaviour. Incorporating more advanced *wearable-specific indicators of PA behaviour* (WIPAB) may be useful when characterising PA profiles and investigating associations with health. We investigated the associations of novel objective measures of PA behaviour with glycated haemoglobin (HbA1c) and insulin sensitivity (Quicki index).

**Methods:**

This observational study included 1026 adults (55% women) aged 18-79y who were recruited from the general population in Luxembourg. Participants provided ≥ 4 valid days of triaxial accelerometry data which was used to derive WIPAB variables related to the activity intensity, accumulation pattern and the temporal correlation and regularity of the acceleration time series.

**Results:**

Adjusted general linear models showed that more time spent in MVPA and a higher average acceleration were both associated with a higher insulin sensitivity. More time accumulated in sedentary behaviour was associated with lower insulin sensitivity. With regard to WIPAB variables, parameters that were indicative of higher PA intensity, including a shallower intensity gradient and higher average accelerations registered during the most active 8 h and 15 min of the day, were associated with higher insulin sensitivity. Results for the power law exponent alpha, and the proportion of daily time accumulated in sedentary bouts > 60 min, indicated that activity which was characterised by long sedentary bouts was associated with lower insulin sensitivity. A greater proportion of time spent in MVPA bouts > 10 min was associated with higher insulin sensitivity. A higher scaling exponent alpha at small time scales (< 90 min), which shows greater correlation in the acceleration time series over short durations, was associated with higher insulin sensitivity. When measured over the entirety of the time series, metrics that reflected a more complex, irregular and unpredictable activity profile, such as the sample entropy, were associated with lower HbA1c levels and higher insulin sensitivity.

**Conclusion:**

Our investigation of novel WIPAB variables shows that parameters related to activity intensity, accumulation pattern, temporal correlation and regularity are associated with insulin sensitivity in an adult general population.

**Supplementary Information:**

The online version contains supplementary material available at 10.1186/s40798-022-00541-9.

## Key points

- Many wearable-specific indicators of physical activity behaviour (WIPAB) were associated with insulin sensitivity (Quicki index) in an adult general population.
- Insulin sensitivity appears to be more sensitive to differences in physical activity than glycated haemoglobin (HbA1c).
- Using WIPAB enables the investigation of new features of physical activity that may be useful when investigating relationships with health.

## Background

Worldwide, one in nine deaths among adults aged 20–79 years is attributable to diabetes, which increased in prevalence by 88% between 2006 and 2019 [1]. In addition to a significant mortality and morbidity burden, a high prevalence of diabetes and prediabetes incurs huge economic cost [2]. Successful strategies to prevent the diabetic state are imperative.

Regular physical activity (PA) is believed to be beneficially associated with insulin sensitivity [3], whereas prolonged and uninterrupted sedentary behaviour (SB) is detrimental [4–6]. The rise of wearable sensors as research tools has opened the door to a more detailed and objective assessment of PA behaviour, compared to self-reported techniques that have primarily been used to date. The most commonly investigated variables over the last decades—and hereafter called “conventional” variables—have been related to the FITT framework: F (frequency), I (intensity), T (time) and T (type) [7] of activity, such as the energy expenditure or the time spent in MVPA or sedentary time. The framework highlights that for a comprehensive description of PA behaviours multiple dimensions need to be considered. However, it was developed with subjective measures in mind. Now, with the increased potential to extract raw acceleration signals from wearable devices, other features can be explored. For instance, how an individual accumulates active or sedentary time might provide important complementary information to conventional variables [8].

A recent scoping review identified several Wearable Indicators of Physical Activity Behaviour (WIPAB) that can been used to quantify the complex and multidimensional nature of PA behaviour [9]. They belong to three different categories that signify: (1) the activity intensity distribution, (2) the accumulation pattern and (3) the temporal correlation and regularity of activity. Additional research is warranted to investigate these parameters, to aid understanding about their added value and their practical feasibility relative to the conventional variables. The objective of the present study was to investigate the associations of novel WIPAB parameters with HbA1c and insulin sensitivity in a general adult population. We hypothesised that WIPAB metrics would be useful with respect to both describing a complex PA behaviour and for examining associations with insulin sensitivity and HbA1c.

## Methods

### Study Population

The second wave of the “Observation of Cardiovascular Risk Factors in Luxembourg” (ORISCAV-LUX2) study collected data on the prevalence of cardiovascular risk factors from 1558 adult participants between January 2016 and February 2018 [10]. This analysis includes participants aged between 25 and 80 years who wore an accelerometer for a minimum of four days (including at least one weekend day), did not report shift work, completed several questionnaires (for collection of demographic, socio-economic, lifestyle and health data) and attended a physical examination for anthropometric measurements and blood sample collections [10].

The ORISCAV-LUX2 study was approved by the National Research Ethics Committee (N° 201.505/12) and the National Commission for Private Data Protection (CNPD). Participants were informed in full about study details and provided written informed consent, and all methods were carried out in accordance with the Declaration of Helsinki.

### Data Collection

PA behaviours (conventional and WIPAB) were objectively measured using an ActiGraph™ GT3X + 3D-accelerometer (Pensacola, USA). The device was worn on the wrist of the non-dominant hand and was used to collect triaxial acceleration data with a sampling frequency of 30 Hz and a dynamic range of ± 8 g. Participants were asked to wear the device continuously for one week, except when showering and during water activities such as swimming.

HbA1c was measured by means of a high-performance liquid chromatography analyser (Tosoh G8), fasting plasma glucose using the Abbott chemistry analyser (colorimetric technique) and fasting insulin via an Abbott immunology analyser (chemiluminescence technique). The quantitative insulin sensitivity check index (Quicki) was calculated using the following equation: 1/ (log (fasting insulin (µU/mL or mU/L) + log (fasting plasma glucose (mg/dL)) [11].

Alcohol consumption and smoking status were assessed by a questionnaire and included the following categorisations: “non-drinker”, “normal drinker” (1–2 glasses per day), “intermediate drinker” (3–4 glasses per day) or “excessive drinker” (at least 5 glasses per day) and “never smoker”, “former smoker” or “current smoker”, respectively. Education and income were self-reported and were defined as years of full-time education and income (in euro) per month, respectively. Depression was measured by means of the *Center for Epidemiologic Studies Depression Scale* (CES-D) and was defined as a score ≥ 16 [12].

### Acceleration Data Processing

Raw triaxial acceleration data were extracted using the manufacturer’s software (ActiLife version 6.13.3) and were calibrated using the open-source R package *GGIR* (version 2.2–0) [13, 14]. The average acceleration magnitude from all axes corrected for gravity (Euclidean Norm Minus One, ENMO) was calculated and averaged over 5-s epochs. An important assumption is that the accelerometers measure gravity as 1* g* (9.81 m/s^2^); thus, to ensure accuracy in the ENMO statistic, all acceleration sensors were calibrated using an autocalibration function [15]. This function capitalises on data from periods of non-movement, when the vector magnitude of all acceleration components should equal 1 *g*. Values deviating from 1 *g* can be used to generate correction factors for application to the data. Files with post-calibration errors above 0.01 *g* (10 m*g*) were excluded [14]. Furthermore, due to different start times, the first and the last half-days of accelerometer wear were excluded. Thus acceleration data were collected for up to six complete days. Participants with less than four valid days of wear time (minimum 10 h per day), including at least one weekend day, were excluded.

The identification of bouts was based on the approach used in the *GGIR* R package [13]. To avoid misclassification of sedentary bouts, overnight sleep periods were excluded from the physical behaviour analysis. Daytime naps starting between 10 am and 6 pm were, however, kept. All variables that were based on the identification of bouts, therefore, only considered awake periods (including daytime naps).

Total sleep duration (hours/day) was assessed using the validated HDCZA (*Heuristic algorithm looking at Distribution of Change in Z-Angle*) algorithm from the *GGIR* R package [16] and was defined as the difference between waking time and onset of sleep.

#### Conventional PA Variables

The total daily time spent in SB and MVPA were utilised as conventional variables. For the identification of PA intensity levels, the validated cut-point definitions of Hildebrand, van Hees [17] and Hildebrand, Hansen [18] were used. Accordingly, the cut-points differentiating SB, light PA (LPA) and MVPA were 44.8 m*g* and 100.6 m*g*, respectively. The average daily acceleration (m*g*) was also used as conventional variable, as it captures the average volume of the entire PA behaviour [19].

#### Wearable-Specific Indicators of PA Behaviour (WIPAB)

Ten different WIPAB parameters, each previously identified in a scoping review [9] and related either to the activity intensity, accumulation pattern or the temporal correlation and regularity of the acceleration time series, were derived for investigation. A detailed description of all WIPAB variables is available in Table 1.

**Table 1:** Description of the wearable-specific indicators of physical activity behaviour (WIPAB)

WIPAB	Description	Interpretation	References
*Activity intensity*			
Intensity gradient	Description of the activity intensity distributions across 24 h	A less negative (higher) intensity gradient reflects more time accumulated across the entire intensity spectrum	Rowlands, Edwardson [20]
MX metric	Quantification of the average acceleration above which the most active *x* minutes or hours are accumulated	A higher MX metric indicates a more intense PA behaviour for a defined time period	Rowlands, Dawkins [19]
*Accumulation pattern*			
Power law exponent alpha	Description of the bout distributions according to their duration for a given activity intensity	A higher power law exponent alpha indicates the accumulation of a certain activity intensity with a greater proportion of shorter bouts	Fortune, Mundell [41]
Proportion of total time accumulated in bouts longer than *x*	Proportion of time accumulated in bouts longer than a certain length *x*	A higher proportion of the total time accumulated in bouts longer than a certain length *x*, reflects a greater imbalance between the number of bouts and their contribution to the accumulated time at that intensity	Chastin and Granat [8]
Gini index	Description of the bout length distributions for a given activity intensity	Higher values indicate a greater inequality in bout lengths (e.g. a relatively high proportion of long bout lengths that contribute to the activity pattern), whereas lower values reflect an activity pattern with a high number of mainly short bouts of similar length	Chastin and Granat [8] Ortlieb, Dias [42]
*Temporal correlation and regularity in the time series*			
Scaling exponent alpha	Detection of temporal correlations in the activity fluctuations by means of the detrended fluctuation analysis (DFA)	Scaling exponent alpha values below 0.5 indicate that the time series is anti-correlated, a value of 0.5 indicates no correlation (“white noise”), and values above 0.5 indicate a positive correlation in fluctuations. Alpha values around 1 indicate the highest temporal correlation in the activity fluctuations	Hu, Van Someren [43] Hu, Riemersma-van der Lek [21]
Autocorrelation at lag *k*	Quantification of the degree of relationship between observations that are *k* lags apart	Autocorrelations coefficients that are closer to 1 or -1 indicate a stronger positive or negative correlation, respectively. Thus, in case of the 24 h autocorrelation, such values would indicate that the timings of the daily activities match perfectly between days or are the exact opposite	Chen, Wu [44] Merilahti and Korhonen [45] Taibi, Price [46]
Lempel–Ziv complexity (LZC)	Quantification of the diversity of subpatterns as well as the dynamics of change between different subpatterns	Higher LZC values indicate a greater chance of the occurrence of new subpatterns in the numeric sequence and, thus, a more complex temporal behaviour	Aboy, Hornero [22] Paraschiv-Ionescu, Perruchoud [23]
Sample entropy	Quantification of the degree of regularity in a time series by analysing the presence of different subsequences (patterns). Regularity in a time series indicates that similar patterns are repeated across time	Higher sample entropy indicates increased disorder, thus greater complexity, irregularity and unpredictability in a time series. Lower values imply a more regular time series	Hauge, Berle [24] Krane-Gartiser, Henriksen [26] Krane-Gartiser, Asheim [25] Scott, Vaaler [27] Delgado-Bonal and Marshak [47]
Symbolic dynamics	Quantification of the complexity of a time series by grouping defined subsequences into different pattern families according to the number and types of variations from one symbol to the next	The rates of occurrences of the four families, expressed as percentage of the total number of patterns analysed, indicates the complexity of the time series	Porta, Guzzetti [29] Guzzetti, Borroni [28]

Table based on findings from a recent scoping review [9]

The intensity gradient and MX metrics focus on the distribution of activity intensities. The intensity gradient, which reflects the drop in time as PA intensity increases, was calculated in intensity bins of 25 m*g* resolution (0–25, 25–50, …, 3975–4000, > 4000 m*g*). A more negative gradient reflects a steeper drop with less time accumulated at higher intensities. A less negative gradient reflects a shallower drop with more time spread across the entire intensity range [20]. MX metrics represent the average acceleration above which the most active “x” minutes of the day are accumulated. For this study, the “x” of the MX metric was set to 8 h (M8) and 15 min (M0.25) [19]. The intensity gradient and MX statistics were retrieved directly from the *GGIR* R package.

Power law exponent (PLE) alpha and the Gini index each describe the accumulation pattern of different activity intensities. PLE alpha describes the distribution of bouts relative to their duration (lower values indicate a greater proportion of longer bouts). To obtain meaningful results PLE alpha was only calculated if a minimum of three different bout lengths could be identified per intensity. For the proportion of the total time accumulated in bouts longer than *x*, the length* x* was set to 60 and 10 min for SB and MVPA, respectively. The Gini index was calculated based on the approach of the *Ineq* R package. The present study focused on the Gini index for SB as it is the most commonly used application of this measure. Higher values indicate a greater inequality in bout lengths, such that an activity profile is characterised by a greater proportion of longer sedentary bouts.

The scaling exponent alpha, the autocorrelation at lag *k*, the Lempel–Ziv complexity, the sample entropy and the symbolic dynamics approach each describe the temporal correlation or regularity of a time series. To determine the scaling exponent alpha, a detrended fluctuation analysis (DFA) was performed, using the *dfa*-function from the *nonlinearTseries* R package. The time scales ranged from 5 to 360 min (6 h), and the number of segments was set to 100. Additionally, the time scales were split up into time scales from 5 to 90 min and from 120 to 360 min to identify short-term and long-term pattern fluctuations, while omitting the transitional region [21]. Higher values for the scaling exponent indicate that large activity values are likely to be followed by large activity values, or small activity values are likely to be followed by small activity values. For the autocorrelation at lag *k*, *k* was set to 24 h (the statistic therefore reflects the regularity and consistency of activity patterns that are 24 h apart) and was analysed by means of the *acf*-function from the *statistics* R package. The Lempel–Ziv complexity (LZC) analysis first requires a reduction of the raw acceleration time series into a numeric sequence, with each number corresponding to a given PA state (i.e. combination of type, intensity and duration) as defined in the *GGIR* R package (see Additional file 1: Table S1). Furthermore, it is recommended to normalise the complexity measure to obtain a metric that is independent of its sequence length. Therefore, the equation from Aboy, Hornero [22] and Paraschiv-Ionescu, Perruchoud [23] was used. A higher LZC indicates a more diverse activity profile. To calculate sample entropy, a higher value of which indicates increased disorder in a time series, the length of the subsequences *m was* set to 2 and the tolerance interval *r* was set to 0.2 times the standard deviation of the time series analysed [24–27]. This normalisation procedure applied to *r* is used to transform the time series into a series with mean 0 and variance 1. For the analysis, the *SampEn*-function from the *TSEntropies* R package was used. The symbolic dynamics approach required a transformation of the time series into a numeric sequence (symbolic sequence) consisting of numbers from 1 to *n* (here set to six), based on the difference between the minimum and maximum value (m*g*) of the analysed time series [28]. To avoid outliers, the minimum was defined as the mean minus three standard deviations, and the maximum as the mean plus three standard deviations. The numeric sequence was then divided into overlapping subsequences (symbolic patterns) of length *L* (here set to three). All subsequences were then grouped into four different pattern families: 1) A pattern with no variation (0 V, e.g. 333), 2) a pattern with only one variation (1 V, e.g. 331 or 133), 3) a pattern with two like variations, where the three symbols either ascend or descend (2LV, e.g. 641 or 235) and 4) a pattern with two unlike variations, with both ascending and descending progressions (2UV, e.g. 312 or 451). This pattern redundancy reduction strategy aims to group all possible patterns into four categories that are characterised by different frequency contents [29]. In the present study, only the most complex family (the 2UV pattern family) was analysed, because inclusion of all four families into statistical models is not recommended due to their compositional nature.

### Statistical Analysis

Descriptive statistics and PA variables were compared between men and women using a Pearson’s Chi-squared test for categorical variables and a one-way ANOVA or a Kruskal–Wallis test for normally or not normally distributed continuous variables, respectively. Correlations between the PA behaviour variables were analysed using a Spearman correlation analysis and were visualised by a correlation matrix.

Separate general linear models were constructed to investigate the associations between each PA variable (conventional and WIPAB) alone with each outcome (HbA1c and Quicki index). Three different models were fitted for each exposure-outcome pair using the *glm*-function from the *stats* R package: (i) an unadjusted model (Model 1), (ii) a model adjusted for age and sex (Model 2), (iii) a model further adjusted for income, education, alcohol consumption, smoking status, total sleep duration, depression and BMI (Model 3). The finally adjusted model 3 was further stratified by sex to investigate potential differences in associations between men and women.

Multiple imputation with a *multivariate imputation by chained equation* (MICE) approach was used to deal with missing data. A missing at random mechanism was assumed. The best predictors were selected based on their correlation with the outcomes [30] using the *quickpred*-function from the *MICE* R package. In total, 30 datasets with 20 iterations were imputed and checked for plausibility [31]. Each of the imputed datasets was used separately to construct the three statistical models. Coefficients were pooled and confidence intervals were calculated according to Rubin’s rules. All statistical analyses were performed in R (version 3.6.1) using RStudio (version 1.3.1093).

## Results

### Study Population and Participant Characteristics

**Fig. 1:**
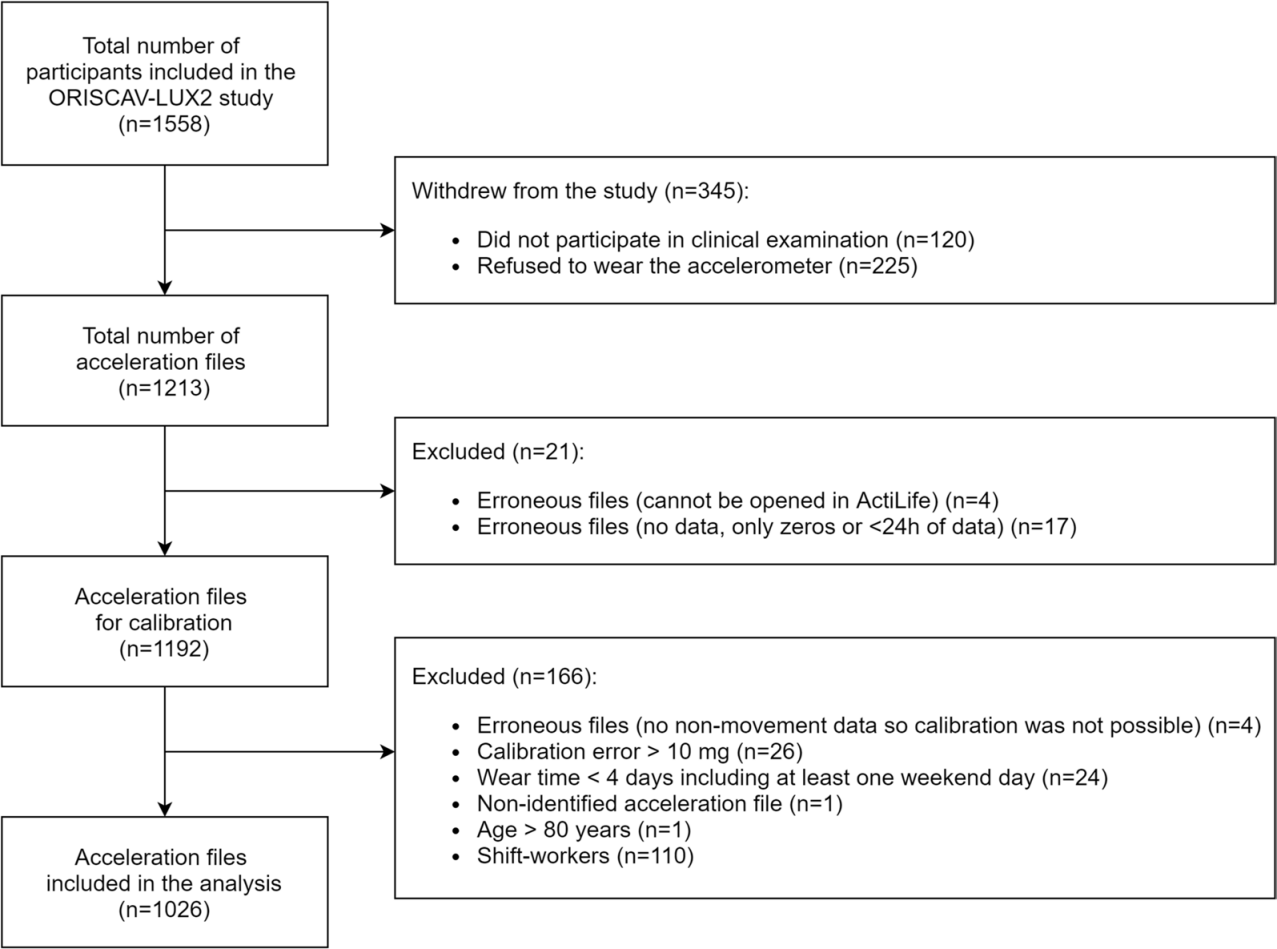
Flow chart of the participants inclusion process

From the 1558 participants included in the ORISCAV-LUX 2 study, 1213 accepted to wear the accelerometer. A total of 75 participants were excluded from the analysis due to: 1) technical issues (e.g. file cannot be opened, no data, *n* = 25), 2) calibration errors above 10 m*g* (*n* = 26) and 3) a too short wear time (*n* = 24). In addition, one file could not be identified, one participant was aged > 80 years and 110 participants reported shift work. Consequently, 1026 participants were included in the present analysis (Fig. 1). Excluded participants were younger (*p* < 0.001), and a greater proportion reported depressive symptoms (*p* < 0.010) and were current smokers (*p* < 0.050), compared to included participants (see Additional file 1: Table S2).

**Fig. 2:**
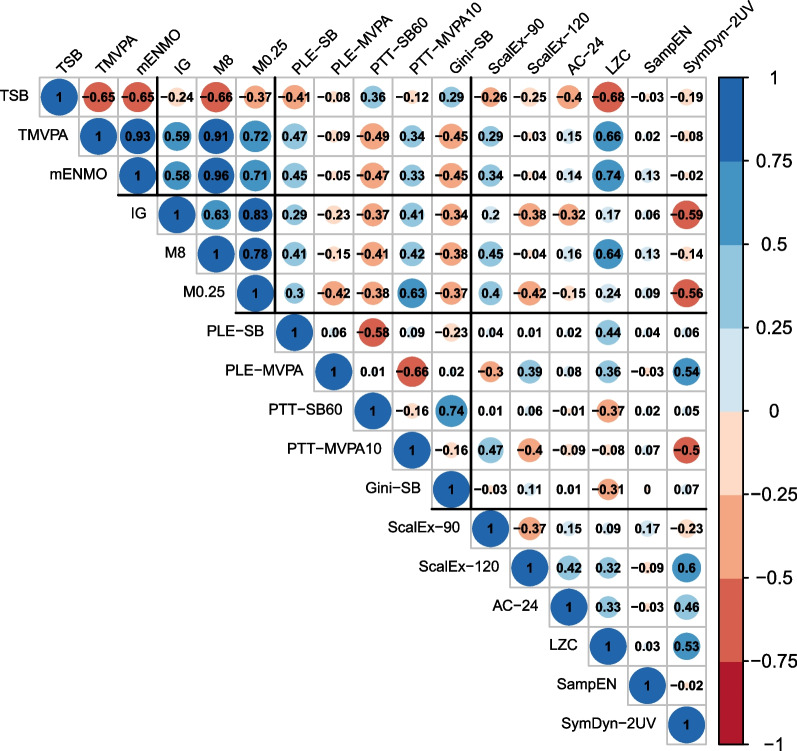
Correlation matrix of the PA behaviour variables. The size of the circle is proportional to the correlation strength. Red and blue colours represent negative and positive correlations, respectively. TSB = Time spent in sedentary behaviour; TMVPA = time spent in moderate to vigorous PA; mENMO = average acceleration; IG = intensity gradient; and M8 = the average acceleration above which the most active 8 h of the day were accumulated; M0.25 = the average acceleration above which the most active 15 min of the day were accumulated; PLE = power law exponent (for SB and MVPA); PTT-SB60 = proportion of total sedentary time accumulated in bouts longer than 60 min; PTT-MVPA10 = proportion of total MVPA time accumulated in bouts longer than 10 min; Gini-SB = Gini index (SB); ScalEx-90 = scaling exponent alpha (< 90 min); ScalEx-120 = scaling exponent alpha (> 120 min); AC-24 = autocorrelation at lag 24 h; LZC = Lempel–Ziv complexity; SampEn = sample entropy; and SymDyn-2UV = symbolic dynamics (2 unlike variations)

The descriptive characteristics of the study population are shown in Table 2. Table 3 summarises the statistics for all PA variables, in the whole sample and stratified by sex. There were several differences in variables between men and women. A correlation matrix of conventional PA and WIPAB variables revealed that each variable was correlated with many others (Fig. 2).

**Table 2:** Descriptive statistics of the study population stratified by sex

Characteristics	All (*n* = 1026) *MED* (*IQR*) or n (%)	Women (*n* = 562) *MED* (*IQR*) or *n* (%)	Men (*n* = 464) *MED* (*IQR*) or *n* (%)	*p* value
Age (years)	51.7 (42.7, 60.4)	52.0 (43.1, 60.1)	51.1 (42.1, 60.7)	0.852
Education (years)	14 (11, 17)	13 (11, 17)	14 (12, 17)	< 0.001*
Income (euro/month)	3571 (2625, 5000)	3333 (2500, 4167)	3750 (2667, 5000)	< 0.010*
Depression (score ≥ 16)	216 (21.1)	142 (25.3)	74 (15.9)	< 0.001*
Marital status				< 0.001*
Single (never married)	118 (11.5)	63 (11.2)	55 (11.9)	
Married/ living with partner	766 (74.7)	394 (70.1)	372 (80.2)	
Divorced/ separated	114 (11.1)	79 (14.1)	35 (7.5)	
Widowed	27 (2.6)	25 (4.4)	2 (0.4)	
Missing	1 (0.1)	1 (0.2)	0 (0.0)	
BMI (kg/m^2^)	25.4 (22.7, 28.6)	24.5 (22.0, 27.7)	26.3 (24.1, 29.3)	< 0.001*
Smoking status				0.066
Never smoker	615 (59.9)	355 (63.2)	260 (56.0)	
Current smoker	121 (11.8)	62 (11.0)	59 (12.7)	
Former smoker	290 (28.3)	145 (25.8)	145 (31.3)	
Alcohol consumption				< 0.001*
Non-drinker	44 (4.3)	28 (5.0)	16 (3.4)	
Normal drinker	697 (67.9)	424 (75.4)	273 (58.8)	
Intermediate drinker	199 (19.4)	79 (14.1)	120 (25.9)	
Excessive drinker	46 (4.5)	7 (1.2)	39 (8.4)	
Missing	40 (3.9)	24 (4.3)	16 (3.4)	
HbA1c (%)	5.40 (5.20, 5.70)	5.40 (5.20, 5.70)	5.40 (5.20, 5.60)	0.153
Quicki index	0.36 (0.34, 0.38)	0.36 (0.34, 0.38)	0.35 (0.33, 0.37)	< 0.001*
Total sleep duration (hours/day)	7.35 (6.64, 7.94)	7.49 (6.87, 8.04)	7.11 (6.42, 7.85)	< 0.001*

*BMI* Body mass index; *HbA1c* glycated haemoglobin; *IQR* interquartile range; and *MED* median

* *p* value < 0.050

**Table 3:** Descriptive statistics of the physical activity behaviour variables stratified by sex

	All (*n* = 1026) *MED* (*IQR*) or mean (*SD*)	Women (*n* = 562) *MED* (*IQR*) or mean (*SD*)	Men (*n* = 464) *MED* (*IQR*) or mean (*SD*)	*p* value
*Conventional variables*				
Time spent in SB (h/day)	12.1 (11.2, 13.1)	11.8 (10.9, 12.7)	12.5 (11.7, 13.5)	< 0.001*
Time spent in MVPA (h/day)	1.33 (0.95, 1.80)	1.36 (0.98, 1.86)	1.29 (0.93, 1.72)	< 0.050*
Average acceleration (m*g*)	25.3 (21.1, 30.1)	26.1 (22.1, 30.6)	24.5 (20.2, 29.5)	< 0.001*
*WIPAB*				
*Activity intensity*				
Intensity gradient	− 2.64 (− 2.77, − 2.51)	− 2.66 (− 2.78, − 2.53)	− 2.62 (− 2.76, − 2.47)	< 0.010*
M8 (m*g*)	44.3 (36.7, 53.1)	45.8 (38.5, 53.5)	42.6 (35.1, 52.9)	< 0.010*
M0.25 (m*g*)	128.3 (101.3, 172.3)	128.0 (101.2, 169.5)	128.4 (101.4, 178.9)	0.466
*Accumulation pattern*				
PLE alpha SB	2.43 (2.29, 2.60)	2.42 (2.30, 2.60)	2.44 (2.28, 2.60)	0.865
PLE alpha MVPA	4.88 (3.52, 7.12)	5.04 (3.73, 7.20)	4.72 (3.36, 6.91)	< 0.050*
Proportions (%)				
SB > 60 min	26.0 (19.1, 33.6)	24.8 (17.2, 32.6)	27.4 (20.7, 35.0)	< 0.001*
MVPA > 10 min	2.93 (0.00, 20.22)	3.00 (0.00, 18.89)	2.83 (0.00, 22.30)	0.546
Gini index SB	0.41 (0.36, 0.46)	0.40 (0.36, 0.45)	0.42 (0.38, 0.47)	< 0.001*
*Temporal correlation and regularity in the time series*				
Scaling exponent alpha				
< 90 min	0.96 (0.91, 1.03)	0.96 (0.92, 1.02)	0.96 (0.90, 1.03)	0.425
> 120 min	0.98 (0.86, 1.07)	0.99 (0.88, 1.08)	0.95 (0.82, 1.06)	< 0.001*
Autocorrelation (lag 24 h)	0.13 (0.10, 0.17)	0.15 (0.11, 0.18)	0.12 (0.09, 0.15)	< 0.001*
LZC†	0.17 (0.04)	0.18 (0.04)	0.16 (0.04)	< 0.001*
Sample entropy	− 0.39 (− 0.64, 0.15)	− 0.37 (− 0.62, 0.14)	− 0.42 (− 0.65, 0.16)	0.785
Symbolic dynamics 2UV (%)	4.21 (3.48, 5.00)	4.55 (3.75, 5.18)	3.91 (3.12, 4.70)	< 0.001*

*LZC* Lempel–Ziv complexity; *MVPA* moderate to vigorous physical activity; *M8* the average acceleration above which the most active 8 h of the day were accumulated; *M0.25* the average acceleration above which the most active 15 min of the day were accumulated; *PLE* power law exponent; *Proportions* proportion of total time accumulated in bouts longer than a certain bout length; *SB* sedentary behaviour; *2UV* two unlike variations; and *WIPAB* wearable-specific indicators of physical activity behaviour

^†^Descriptive statistics presented as mean (*SD*) as the variable is normally distributed; * *p* value < 0.050

### Association of Conventional PA and WIPAB Variables with HbA1c and the Quicki Index

The associations between PA variables with HbA1c and the Quicki index are presented in Table 4. In unadjusted model 1, all variables except the power law exponent alpha (for MVPA) and the LZC were associated with HbA1c levels. After adjustment for age and sex (Model 2), a higher intensity gradient, higher M0.25, and a greater proportion of accumulated MVPA in bouts longer than 10 min, a higher scaling exponent alpha at small time scales (< 90 min) and a higher sample entropy were each associated with lower HbA1c. Conversely, higher percentages of 2UV patterns and more time spent in SB were associated with higher HbA1c levels. After further adjustments for income, education, alcohol consumption, smoking status, total sleep duration, depression and BMI (Model 3), only sample entropy remained significantly associated with lower HbA1c levels.

**Table 4:** Associations of conventional PA and WIPAB variables with HbA1c and the Quicki index, respectively

	HbA1c			Quicki index		
	Model 1	Model 2	Model 3	Model 1	Model 2	Model 3
*Conventional variables*						
Time spent in SB† (h/day)	0.39 (0.20, 0.58)*	0.27 (0.08, 0.46)*	0.06 (− 0.16, 0.29)	− 0.06 (− 0.07, − 0.04)*	− 0.04 (− 0.05,− 0.03)*	− 0.03 (− 0.05, − 0.02)*
Time spent in MVPA (h/day)	− 0.09 (− 0.14, − 0.05)*	− 0.02 (− 0.07, 0.02)	− 0.01 (− 0.05, 0.03)	0.01 (0.01, 0.01)*	0.01 (0.00, 0.01)*	0.00 (0.00, 0.01)*
Average acceleration (*mg*)†	− 0.08 (− 0.12, − 0.04)*	− 0.19 (− 0.06, 0.02)	− 0.08 (− 0.05, 0.03)	0.01 (0.01, 0.01)*	0.01 (0.01, 0.01)*	0.01 (0.00, 0.01)*
*WIPAB*						
*Activity intensity*						
Intensity gradient	− 0.52 (− 0.64, − 0.39)*	− 0.19 (− 0.33, − 0.06)*	− 0.08 (− 0.22, 0.05)	0.04 (0.03, 0.05)*	0.03 (0.03, 0.04)*	0.02 (0.01, 0.03)*
M8 (*mg*)†	− 0.03 (− 0.05, − 0.02)*	− 0.01 (− 0.03, 0.00)	− 0.00 (− 0.02, 0.01)	0.01 (0.00, 0.01)*	0.00 (0.00, 0.01)*	0.00 (0.00, 0.00)*
M0.25 (*mg*)†	− 0.01 (− 0.01, − 0.01)*	− 0.00 (− 0.01, − 0.00)*	− 0.00 (− 0.00, 0.00)	0.00 (0.00, 0.00)*	0.00 (0.00, 0.00)*	0.00 (0.00, 0.00)*
*Accumulation pattern*						
PLE alpha SB	− 0.26 (− 0.38, − 0.14)*	− 0.03 (− 0.15, 0.09)	0.01 (− 0.10, 0.13)	0.02 (0.02, 0.03)*	0.02 (0.01, 0.03)*	0.01 (0.00, 0.02)*
PLE alpha MVPA††	0.56 (− 0.25, 1.36)	0.40 (− 0.34, 1.14)	− 0.08 (− 0.82, 0.66)	− 0.05 (− 0.10, − 0.01)*	− 0.06 (− 0.10, − 0.01)*	0.00 (− 0.04, 0.04)
Proportions (%)						
SB > 60 min	0.56 (0.31, 0.82)*	0.12 (− 0.14, 0.37)	− 0.07 (− 0.32, 0.18)	− 0.06 (− 0.08, − 0.05)*	− 0.04 (− 0.06, − 0.02)*	− 0.02 (− 0.03, 0.00)*
MVPA > 10 min	− 0.18 (− 0.34, − 0.03)*	− 0.19 (− 0.33, − 0.04)*	− 0.08 (− 0.22, 0.06)	0.03 (0.02, 0.04)*	0.04 (0.02, 0.05)*	0.02 (0.01, 0.03)*
Gini index SB	0.84 (0.44, 1.25)*	0.25 (− 0.15, 0.64)	− 0.01 (− 0.40, 0.38)	− 0.09 (− 0.12, − 0.06)*	− 0.05 (− 0.08, − 0.03)*	− 0.02 (− 0.04, 0.01)
*Temporal correlation and regularity in the time series*						
Scaling exponent alpha						
< 90 min	− 0.33 (− 0.64, − 0.03)*	− 0.42 (− 0.70, − 0.14)*	− 0.25 (− 0.53, 0.03)	0.06 (0.04, 0.08)*	0.06 (0.04, 0.08)*	0.04 (0.02, 0.06)*
> 120 min	0.25 (0.06, 0.45)*	0.07 (− 0.11, 0.26)	0.02 (− 0.18, 0.21)	− 0.02 (− 0.03, 0.00)*	− 0.02 (− 0.03, 0.00)*	− 0.01 (− 0.02, 0.00)
Autocorrelation (lag 24 h)	1.00 (0.49, 1.51)*	0.26 (− 0.24, 0.76)	0.35 (− 0.16, 0.85)	0.00 (− 0.04, 0.03)	0.00 (− 0.04, 0.03)	0.00 (− 0.04, 0.03)
LZC	− 0.66 (− 1.37, 0.05)	0.09 (− 0.59, 0.78)	− 0.08 (− 0.78, 0.61)	0.14 (0.09, 0.19)*	0.07 (0.03, 0.12)*	0.07 (0.03, 0.12)*
Sample entropy††	− 5.56 (− 8.92, − 2.21)*	− 5.15 (− 8.26, − 2.04)*	− 4.73 (− 7.77, − 1.69)*	0.28 (0.04, 0.51)*	0.30 (0.07, 0.53)*	0.22 (0.03, 0.42)*
Symbolic dynamics 2UV (%)	5.73 (3.46, 8.01)*	2.64 (0.37, 4.91)*	0.34 (− 1.98, 2.67)	− 0.32 (− 0.48, − 0.16)*	− 0.40 (− 0.56, − 0.23)*	− 0.15 (− 0.30, 0.00)*

Values are presented as coefficients (95% CI), which were calculated according to Rubin’s rule. All models were performed with imputed data

Model 1: unadjusted model; Model 2: adjusted for age and sex; Model 3: model 2 further adjusted for income, education, alcohol consumption, smoking status, total sleep duration, depression and BMI

*HbA1c* glycated haemoglobin; *LZC* Lempel–Ziv complexity; *MVPA* moderate to vigorous physical activity; *M8* the average acceleration above which the most active 8 h of the day were accumulated; *M0.25* the average acceleration above which the most active 15 min of the day were accumulated; *PLE* power law exponent; *Proportions* proportion of total time (SB time or MVPA time, respectively) accumulated in bouts longer than a certain bout length; *SB* sedentary behaviour; and *2UV* two unlike variations

^†^Variable scaled by dividing by 10

^††^ Variables scaled by dividing by 100

* *p* value < 0.050

Except for the autocorrelation at lag 24 h, all PA behaviour variables were associated with the Quicki index in statistical models 1 (unadjusted) and 2 (adjusted for age and sex). In finally adjusted model 3, more time accumulated in MVPA, a greater proportion of MVPA accumulated in bouts longer than 10 min and higher values for the average acceleration, intensity gradient, M0.25 and M8, were each associated with a higher Quicki index. Additionally, a higher power law exponent alpha for SB, higher scaling exponent alpha at small time scales (< 90 min) and a higher LZC and higher sample entropy were associated with a higher Quicki index. In contrast, more time accumulated in SB, a higher proportion of SB time accumulated in bouts longer than 60 min and higher percentages of 2UV patterns were associated with lower Quicki index.

Results stratified by sex are provided in the Additional file (see Additional file 1: Table S3). There was considerable overlap of most confidence intervals, but the scaling exponent alpha (< 90 min) was significantly associated with lower HbA1c only in women, and a higher sample entropy was associated with lower HbA1c only in men. The proportion of SB time accumulated in bouts longer than 60 min, the power law exponent alpha for SB, Gini index, LZC and the sample entropy were significantly associated with insulin sensitivity only in women. Higher percentages of 2UV patterns were associated with lower Quicki index but only in men.

## Discussion

The main aim of this study was to investigate associations of PA behaviours with HbA1c and insulin sensitivity in a general adult population using WIPAB. We hypothesised that novel WIPAB measures would be useful when characterising PA, which is a complex and multifaceted behaviour, and may provide new information about the associations of PA with insulin sensitivity and HbA1c. We identified that a higher sample entropy, which is a marker of the complexity of behaviour, was independently associated with lower HbA1c levels and higher insulin sensitivity. In addition, all WIPAB parameters from the intensity distribution category (the intensity gradient, M8 and M0.25 metrics), total MVPA, the proportion of MVPA time that was accumulated in bouts longer than 10 min, the power law exponent alpha (for SB), the scaling exponent alpha at small time scales and the LZC were favourably associated with insulin sensitivity. In contrast, higher proportions of SB time accumulated in bouts longer than 60 min and higher percentages of 2UV patterns were associated with lower insulin sensitivity.

We found high correlations between many of the conventional PA and WIPAB indicators, which highlights that caution is warranted when building multivariable regression models that include several PA indicators. The findings may also point towards a certain redundancy of some variables. For instance, conventional PA variables and WIPAB parameters from the intensity distribution category were highly correlated, confirming to some extent an overlap of features. However, they could also complement each other. Our findings highlight the need to use more advanced statistical methods than simple regression models, which are generally used to investigate associations of only one PA component at a time with outcomes. Alternative approaches such as unsupervised clustering methods (e.g. k-means or hierarchical agglomerative clustering) or dimensionality reduction methods (e.g. principal component analysis) could be used to combine information from conventional PA and WIPAB variables. This may enable identification of PA behaviour patterns, more sophisticated assessment of the complexity of human PA behaviour in real-life settings and greater precision in studying its relation with various health outcomes.

We found that each WIPAB variable from the intensity distribution category, more time spent in MVPA (in total and in bouts of at least 10 min) and a higher average acceleration across 24 h were each beneficially associated with insulin sensitivity. This is in agreement with the observations of previous studies, which have shown that total PA [32, 33], increasing PA intensity [34] and more time spent in MVPA [33, 35] are favourably associated with markers of insulin sensitivity. One study reported that the total number of activity counts per day exhibited stronger associations compared to MVPA. The authors hypothesised that on top of MVPA accumulated in bouts, total counts also captures LPA and intermittent MVPA [32]. Indeed, some investigations have found that LPA [34, 36] and short bouts (one to nine minutes) of MVPA [33] are beneficially associated with insulin sensitivity. A meta-analysis of controlled trials also indicated that compared to one continuous energy-matched PA episode, frequent PA bouts may be more beneficial for postprandial glucose levels [5]. Each of these observations supports the proposition that a more all-encompassing and comprehensive assessment of PA could be advantageous for the investigation of health-related outcomes.

We found that more time accumulated in SB was associated with lower insulin sensitivity, but not with HbA1c levels. A recent meta-analysis of free-living interventions that targeted reductions in SB also identified more evidence for intervention effects when fasting insulin was the outcome of interest, as opposed to HbA1c [4]. This might be because changes in insulin sensitivity precede increases in HbA1c [37], which is a longer-term marker of chronic glucose dysregulation. With regard to accumulation patterns, meta-analyses of controlled trials have shown that breaking-up prolonged and uninterrupted sedentary behaviour with PA attenuates postprandial blood glucose and insulin levels [5, 6]. In line with these findings, we observed that a greater proportion of SB time that was accumulated in long bouts lasting at least 60 min, and a higher Gini index, were both adversely associated with insulin sensitivity, most notably in women. Also in women, a higher power law exponent alpha (which reflects a greater proportion of shorter SB bouts) was favourably associated with insulin sensitivity. The apparent sex differences are supported by two trials, which interrupted prolonged sitting with brief walking bouts (performed every 20–30 min), and which reported that women experienced greater reductions in postprandial glucose concentration than men [38, 39]. It appears that fractionalised SB might contribute to improved glucose control in women. Future studies should explore mechanisms that may explain the observed sex difference in associations.

We found that a higher scaling exponent alpha, which indicates a stronger positive correlation in activity fluctuations across small time scales (< 90 min), was associated with higher insulin sensitivity. So too was higher complexity in the time series, as indicated by the results for LZC and sample entropy, particularly for women. Is it noteworthy that sample entropy, which was incidentally the only parameter that was associated with both insulin sensitivity and HbA1c, was weakly correlated with all other PA variables. A previous study reported that older adults who were highly active exhibited a more complex activity pattern and a higher sample entropy relative to adults who were less active [40]. We also observed that physically active participants (who accumulated ≥ 150 min MVPA/week in > 10 min bouts) in our sample displayed a higher entropy than inactive participants (− 0.26 (− 0.67, 0.18) vs. − 0.40 (− 0.64, 0.14, *p* = 0.003). The results imply that active people have more unpredictable activity patterns. There may be a rationale for investigating the potential benefits on glycaemic control of variable PA routines, and the relative disadvantages of structured and predictable schedules [40]. In contrast to the results for LZC and sample entropy, higher proportions of 2UV patterns were associated with lower insulin sensitivity, most notably in men. It is possible that the symbolic dynamics approach captures a different aspect of the complexity of PA behaviour, one which may be complementary to the features of other complexity markers.

The use of novel WIPAB parameters, which enable the investigation of new features of PA behaviour across multiple dimensions, may prove to have important clinical and public health implications. Indeed, we have found that several WIPAB variables were associated with insulin sensitivity. It is important to consider, however, that although we observed several statistically significant associations, how they translate to the prevention of prediabetes and diabetes remains unclear. Several of the WIPAB variables are complex, potentially difficult to comprehend, and do not lend themselves to straightforward interpretation. Researchers will have to decide if they prefer potentially more accurate and comprehensive PA behaviour metrics that are less interpretable, or simpler and more intuitive metrics. Either way, prospective studies are needed to investigate which features of PA behaviour might be decisive for the development and progression of important health conditions, including diabetes.

### Strengths and Limitations

To our knowledge, this is the first study to investigate multiple domains of PA behaviour, in order to identify which parameters are associated with insulin sensitivity and HbA1c. We capitalised on a large study sample, objective measurements of PA behaviour, and performed an in-depth analysis that included adjustment for a diverse range of relevant confounding factors. It should be noted, however, that the cross-sectional design of the study does not allow any conclusions on causality. Future longitudinal studies are needed to investigate the associations of novel WIPAB parameters with diabetes-related outcomes.

## Conclusions

Nearly all of the WIPAB variables were associated with insulin sensitivity in an adult general population. This demonstrates that WIPAB parameters may be useful when analysing the associations of PA with health-related factors. Longitudinal studies are needed to investigate which precise features of PA are important for diabetes prevention.

## Supplementary Information

**Additional file 1. Table S1:** Definition of the PA states in the GGIR package [13]. **Table S2:** Comparison between included and excluded participants. **Table S3:** Stratified analyses by sex for HbA1c and the Quicki index, using the finally adjusted model.

## Data Availability

Data are available upon reasonable request to LM. De-identified participant data might be available after the consent of all authors and the ORISCAV study group. Requests to access the dataset should be directed to Laurent Malisoux, laurent.malisoux@lih.lu.

## Abbreviations

BMI: Body mass index
CES-D: Center for Epidemiologic Studies Depression Scale
CI: Confidence interval
CNPD: National Commission for Private Data Protection
DFA: Detrended fluctuation analysis
FITT framework: Frequency, intensity, time, type of physical activity
HbA1c: Glycated haemoglobin
HDCZA: Heuristic algorithm looking at Distribution of Change in Z-Angle
IQR: Interquartile range
LPA: Light physical activity
LZC: Lempel–Ziv complexity
MED: Median
MICE: Multivariate imputation by chained equation
MVPA: Moderate to vigorous physical activity
M0.25: Average acceleration above which the most active 15 min of the day were accumulated
M8: Average acceleration above which the most active 8 h of the day were accumulated
ORISCAV-LUX 2 study: Second wave of the “Observation of Cardiovascular Risk Factors in Luxembourg” study
PA: Physical activity
PLE alpha: Power law exponent alpha
Quicki: Quantitative insulin sensitivity check index
SB: Sedentary behaviour
SD: Standard deviation
WIPAB: Wearable-specific indicators of physical activity behaviour
0 V: No variation (symbolic dynamics approach)
1 V: One variation (symbolic dynamics approach)
2LV: Two like variations (symbolic dynamics approach)
2UV: Two unlike variations (symbolic dynamics approach)
