# Supplementary material for: Associations Between Wearable-Specific Indicators of Physical Activity Behaviour and Insulin Sensitivity and Glycated Haemoglobin in the General Population: Results from the ORISCAV-LUX 2 Study
Source: Sports Med Open. 2022 Dec 12;8:146. doi: 10.1186/s40798-022-00541-9 (PMC9743939; doi:10.1186/s40798-022-00541-9)
Supplement: Supplementary file 1 — Additional file 1. Table S1: Definition of the PA states in the GGIR package [13]. Table S2: Comparison between included and excluded participants. Table S3: Stratified analyses by sex for HbA1c and the Quicki index, using the finally adjusted model. [file 40798_2022_541_MOESM1_ESM.docx]

**Supplementary material**

**Table S1.** Definition of the PA states in the GGIR package (Migueles et al., 2019).

| **Type** | **Intensity** | **Duration** | **PA state** | |
| --- | --- | --- | --- | --- |
| Sleep period time – sleep |  |  | 0 | a |
| Sleep period time – wakefulness | Inactivity/ minimal movement  (acc ≤ 44.8 m*g*) |  | 1 | b |
|  | Light activity  (44.8 < acc ≤ 100.6 m*g*) |  | 2 | c |
|  | Moderate activity  (100.6 < acc ≤ 428.8 m*g*) |  | 3 | d |
|  | Vigorous activity  (acc > 428.8 m*g*) |  | 4 | e |
| Daytime | Inactivity/ minimal movement  (acc ≤ 44.8 m*g*) | < 10 min (*very short*) | 5 | f |
|  | Light activity  (44.8 < acc ≤ 100.6 m*g*) | < 1 min (*very short*) | 6 | g |
|  | Moderate activity  (100.6 < acc ≤ 428.8 m*g*) |  | 7 | h |
|  | Vigorous activity  (acc > 428.8 m*g*) |  | 8 | i |
|  | Moderate to vigorous activity (acc > 100.6 m*g*) | ≥ 10 min (*long*) | 9 | j |
|  |  | 5 ≤ duration < 10 (*medium*) | 10 | k |
|  |  | 1 ≤ duration < 5 (*short*) | 11 | l |
|  | Inactivity/ minimal movement (acc ≤ 44.8 m*g*) | ≥ 30 min (*long*) | 12 | m |
|  |  | 20 ≤ duration < 30 (*medium*) | 13 | n |
|  |  | 10 ≤ duration < 20 (*short*) | 14 | o |
|  | Light activity  (44.8 < acc ≤ 100.6 m*g*) | ≥ 10 min (*long*) | 15 | p |
|  |  | 5 ≤ duration < 10 (*medium*) | 16 | q |
|  |  | 1 ≤ duration < 5 (*short*) | 17 | r |

Abbreviations: acc = acceleration, m*g* = milligravity (gravitational unit).

**Table S2.** Comparison between included and excluded participants.

| **Characteristics** | **Included (n=1026)**  *MED* (*IQR*) or *n* (%) | **Excluded (n=532)**  *MED* (*IQR*) or *n* (%) | ***p*-value** |
| --- | --- | --- | --- |
| Sex |  |  | 0.051 |
| Female | 562 (54.8) | 263 (49.4) |  |
| Male | 464 (45.2) | 269 (50.6) |  |
| Age (years) | 51.7 (42.7, 60.4) | 48.0 (39.3, 56.8) | < 0.001* |
| Education (years) | 14 (11, 17) | 14 (11, 17) | 0.437 |
| Income (euro/month) | 3571 (2625, 5000) | 3452 (2019, 4348) | 0.054 |
| Depression (score ≥ 16) | 216 (21.1) | 143 (26.9) | < 0.010* |
| Marital status |  |  | 0.607 |
| Single (never married) | 118 (11.5) | 70 (13.2) |  |
| Married/ living with partner | 766 (74.7) | 389 (73.1) |  |
| Divorced/ separated | 114 (11.1) | 62 (11.7) |  |
| Widowed | 27 (2.6) | 10 (1.9) |  |
| Missing | 1 (0.1) | 1 (0.2) |  |
| BMI (kg/m^2^) | 25.4 (22.7, 28.6) | 25.6 (23.1, 29.0) | 0.379 |
| Smoking status |  |  | < 0.050* |
| Never smoker | 615 (59.9) | 293 (55.1) |  |
| Current smoker | 121 (11.8) | 89 (16.7) |  |
| Former smoker | 290 (28.3) | 133 (25.0) |  |
| Alcohol consumption |  |  | 0.447 |
| Non-drinker | 44 (4.3) | 30 (5.6) |  |
| Normal drinker | 697 (67.9) | 347 (65.2) |  |
| Intermediate drinker | 199 (19.4) | 91 (17.1) |  |
| Excessive drinker | 46 (4.5) | 27 (5.1) |  |
| Missing | 40 (3.9) | 37 (7.0) |  |
| HbA1c (%) | 5.40 (5.20, 5.70) | 5.40 (5.20, 5.70) | 0.469 |
| Quicki index | 0.36 (0.34, 0.38) | 0.35 (0.33, 0.38) | 0.297 |

Abbreviations: BMI = Body mass index; HbA1c = glycated haemoglobin; IQR = interquartile-range; MED = median.

* *p*-value < 0.050.

**Table S3.** Stratified analyses by sex for HbA1c and the Quicki index, using the finally adjusted model.

|  | **HbA1c** | | **Quicki index** | |
| --- | --- | --- | --- | --- |
|  | **Women (n=562)** | **Men (n=464)** | **Women (n=562)** | **Men (n=464)** |
| ***Conventional variables*** |  |  |  |  |
| Time spent in SB† (h) | 0.10 (-0.19, 0.38) | 0.00 (-0.36, 0.36) | -0.03 (-0.05, -0.01)* | -0.03 (-0.05, -0.01)* |
| Time spent in MVPA (h) | -0.01 (-0.06, 0.04) | -0.02 (-0.09, 0.05) | 0.00 (0.00, 0.01)* | 0.01 (0.00, 0.01)* |
| Average acceleration (*mg*)† | -0.01 (-0.06, 0.04) | -0.01 (-0.07, 0.05) | 0.01 (0.00, 0.01)* | 0.01 (0.00, 0.01)* |
|  |  |  |  |  |
| ***WIPAB*** |  |  |  |  |
| *Activity intensity* |  |  |  |  |
| Intensity gradient | -0.14 (-0.34, 0.05) | -0.05 (-0.24, 0.15) | 0.02 (0.00, 0.03)* | 0.02 (0.01, 0.03)* |
| M8 (*mg*)† | -0.00 (-0.02, 0.02) | -0.01 (-0.03, 0.02) | 0.00 (0.00, 0.00)* | 0.00 (0.00, 0.01)* |
| M0.25 (*mg*)† | -0.00 (-0.01, 0.00) | -0.00 (-0.01, 0.00) | 0.00 (0.00, 0.00)* | 0.00 (0.00, 0.00)* |
| *Accumulation pattern* |  |  |  |  |
| PLE alpha SB | -0.01 (-0.17, 0.14) | 0.04 (-0.14, 0.22) | 0.01 (0.00, 0.03)* | 0.01 (0.00, 0.02) |
| PLE alpha MVPA†† | -0.34 (-1.28, 0.60) | 0.27 (-0.83, 1.37) | 0.00 (-0.06, 0.06) | -0.01 (-0.06, 0.05) |
| Proportions (%) |  |  |  |  |
| SB > 60 min | 0.11 (-0.22, 0.44) | -0.26 (-0.64, 0.12) | -0.03 (-0.06, -0.01)* | 0.00 (-0.02, 0.02) |
| MVPA > 10 min | -0.08 (-0.28, 0.12) | -0.11 (-0.31, 0.10) | 0.02 (0.00, 0.03)* | 0.03 (0.02, 0.04)* |
| Gini index SB | 0.35 (-0.17, 0.87) | -0.40 (-1.00, 0.21) | -0.04 (-0.07, 0.00)* | 0.00 (-0.03, 0.04) |
| *Temporal correlation and regularity in the time-series* |  |  |  |  |
| Scaling exponent alpha |  |  |  |  |
| < 90 min | -0.43 (-0.84, -0.01)* | -0.10 (-0.49, 0.29) | 0.03 (0.00, 0.06)* | 0.05 (0.03, 0.07)* |
| > 120 min | 0.04 (-0.23, 0.31) | 0.00 (-0.27, 0.27) | 0.00 (-0.02, 0.02) | -0.01 (-0.03, 0.00) |
| Autocorrelation (lag 24h) | 0.09 (-0.58, 0.77) | 0.56 (-0.21, 1.33) | 0.00 (-0.05, 0.05) | 0.00 (-0.05, 0.04) |
| LZC | -0.38 (-1.32, 0.55) | 0.28 (-0.77, 1.32) | 0.10 (0.03, 0.16)* | 0.05 (-0.01, 0.11) |
| Sample entropy†† | -1.99 (-6.04, 2.06) | -7.59 (-12.23, -2.94)* | 0.32 (0.04, 0.61)* | 0.09 (-0.18, 0.37) |
| Symbolic dynamics 2UV (%) | -0.56 (-3.78, 2.66) | 1.68 (-1.73, 5.08) | -0.08 (-0.31, 0.14) | -0.26 (-0.45, -0.06)* |

Values are presented as coefficients (95% CI), which were calculated according to Rubin’s rule. All models were performed with imputed data and included income, education, alcohol consumption, smoking status, total sleep duration, depression and BMI as confounder. Abbreviations: HbA1c = glycated haemoglobin; LZC = Lempel-Ziv complexity; MVPA = moderate to vigorous physical activity; M8 = the average acceleration above which the most active 8 hours of the day were accumulated; M0.25 = the average acceleration above which the most active 15 min of the day were accumulated; PLE = power law exponent; Proportions = proportion of total time accumulated in bouts longer than a certain bout length; SB = sedentary behaviour; 2UV = two unlike variations.

† Variable scaled by dividing by 10; †† Variables scaled by dividing by 100; * *p*-value < 0.050.

**Reference**

Migueles, JH, Rowlands, AV, Huber, F, Sabia, S, & van Hees, VT. (2019). GGIR: A research community–driven open source R package for generating physical activity and sleep outcomes from multi-day raw accelerometer data. *J Meas Phys Behav, 2*(3), 188-196.
